# Neuropilin2 in Mesenchymal Stromal Cells as a Potential Novel Therapeutic Target in Myelofibrosis

**DOI:** 10.3390/cancers16101924

**Published:** 2024-05-18

**Authors:** Karla Vosbeck, Sarah Förster, Thomas Mayr, Anshupa Sahu, El-Mustapha Haddouti, Osamah Al-Adilee, Ruth-Miriam Körber, Savita Bisht, Michael H. Muders, Svetozar Nesic, Andreas Buness, Glen Kristiansen, Frank A. Schildberg, Ines Gütgemann

**Affiliations:** 1 Institute for Pathology, University Hospital Bonn, 53127 Bonn, Germanythomas.mayr@ukbonn.de (T.M.); s4osalad@uni-bonn.de (O.A.-A.); muderslab@gmail.com (M.H.M.); glen.kristiansen@ukbonn.de (G.K.); 2 Institute for Medical Biometry, Informatics and Epidemiology, Medical Faculty, University of Bonn, 53127 Bonn, Germany; anshupa.vssut@gmail.com; 3 Department of Orthopedics and Trauma Surgery, University Hospital Bonn, 53127 Bonn, Germany; haddouti@googlemail.com (E.-M.H.);; 4 Department of Medicine III, University Hospital Bonn, 53127 Bonn, Germany; ruth-miriam.koerber@ukbonn.de (R.-M.K.); savita.bisht@ukbonn.de (S.B.); 5 Core Unit for Bioinformatics Data Analysis, Medical Faculty, University of Bonn, Venusberg-Campus 1, 53127 Bonn, Germany; nesic@uni-bonn.de (S.N.); andreas.buness@uni-bonn.de (A.B.)

**Keywords:** myeloproliferative neoplasm, myelofibrosis, mesenchymal stromal cells, neuropilin 2, endosteal niche

## Abstract

**Simple Summary:**

Bone marrow (myelo-) fibrosis in myeloproliferative neoplasms is associated with poor prognosis and treatment failure emphasizing the importance of investigating novel treatment approaches outside the JAK pathway. This study investigates the role of neuropilin 2 (NRP2) in myelofibrosis and bone formation, identifies the type of stromal cells expressing and upregulating NRP2 and outcomes when NRP2 is lost. Our results suggest that NRP2 is an interesting molecular druggable target in myeloproliferative neoplasm.

**Abstract:**

Bone marrow fibrosis in myeloproliferative neoplasm (MPN), myelodysplastic syndromes (MDS), MPN/MDS overlap syndromes and acute myeloid leukemia (AML) is associated with poor prognosis and early treatment failure. Myelofibrosis (MF) is accompanied by reprogramming of multipotent bone marrow mesenchymal stromal cells (MSC) into osteoid and fiber-producing stromal cells. We demonstrate NRP2 and osteolineage marker NCAM1 (neural cell adhesion molecule 1) expression within the endosteal niche in normal bone marrow and aberrantly in MPN, MDS MPN/MDS overlap syndromes and AML (*n* = 99), as assessed by immunohistochemistry. Increased and diffuse expression in mesenchymal stromal cells and osteoblasts correlates with high MF grade in MPN (*p* < 0.05 for NRP2 and NCAM1). Single cell RNA sequencing (scRNAseq) re-analysis demonstrated NRP2 expression in endothelial cells and partial co-expression of NRP2 and NCAM1 in normal MSC and osteoblasts. Potential ligands included transforming growth factor β1 (TGFB1) from osteoblasts and megakaryocytes. Murine ThPO and JAK2*^V617F^* myelofibrosis models showed co-expression of Nrp2 and Ncam1 in osteolineage cells, while fibrosis-promoting MSC only express Nrp2. In vitro experiments with MC3T3-E1 pre-osteoblasts and analysis of *Nrp2^−^*^/*−*^ mouse femurs suggest that Nrp2 is functionally involved in osteogenesis. In summary, NRP2 represents a potential novel druggable target in patients with myelofibrosis.

## 1. Introduction

Bone marrow (BM) myelofibrosis is characterized by increased deposition of extracellular matrix proteins, such as collagen and reticulin fibers by mesenchymal stromal cells (MSC). It is observed in primary myelofibrosis (PMF), but also occurs in other myeloproliferative (MPN) and myeloid neoplasms (myelodysplastic syndromes (MDS), MPN/MDS overlap syndromes and secondary AML). Especially, PMF is often accompanied by osteosclerosis (increased bone formation preceded by increased collagen) and a profibrotic osteosclerotic stroma can be ameliorated by risk-adapted therapy, especially JAK2 inhibition [1,2]. However, JAK2 inhibition is non-curative, has side effects [3], and resistance to JAK2 inhibitors is common, calling for new therapeutic approaches outside the JAK-STAT pathway [4]. 

In murine JAK2*^V617F^*-driven [5] and ThPO lentiviral models [6] of myelofibrosis, multipotent Cxcl12^+^ MSC differentiate into collagen-producing, Gli1^+^ myofibroblast-like cells [7] and osteolineage cells [8]. Targeting myelofibrosis seems to be particularly attractive in PMF, as here myelofibrosis is most rapidly progressing. Additionally, in PMF, myelofibrosis is most commonly accompanied by osteosclerosis.

In our search for molecular druggable targets acting on MSC in myelofibrotic stroma, we focused our interest on neuropilin receptors (NRPs), signal modulating co-receptors for members of the family of vascular endothelial growth factors (VEGFs), class 3 semaphorins and TGFβ [9]. NRP1 and NRP2 are structurally similar cell surface proteins that stimulate angiogenesis, lymph-angiogenesis, immune tolerance, and are involved in tumorigenesis and metastasis [10,11,12,13,14].

First, we assessed NRP2 immunohistochemical expression patterns in BM trephine biopsies of myelofibrosis patients in situ. We selected NCAM1 as a marker to localize osteogenic lineage cells in situ, as it is expressed in osteoblasts and is functionally involved in osteogenic differentiation and repair [15,16,17]. Interestingly, we observe NCAM1 and NRP2 colocalization in the endosteal niche in sparse endosteal stromal cells in normal BM that increased in myeloid neoplasms associated with fibrosis. To further dissect the impact of NRP2 on fibrogenesis and osteogenesis, we re-analyzed scRNAseq BM atlas data focusing on NRP2 ligand and receptor expression in normal and fibrosed BM MSCs. Last, we aimed to investigate the functional role of NRP2 during osteogenic differentiation using murine pre-osteoblast cell line MC3T3-E1 [18] in vitro and *Nrp2*^−/−^ mice [19] in vivo.

## 2. Materials and Methods

### 2.1. Patient Cohort

This retrospective study was performed using archived formalin-fixed, paraffin-embedded BM biopsies from 122 patients. BM biopsies collected between 2013 and 2023 were identified from the digital archive and the MPN outpatient ward of the University Hospital Bonn. Analysis was performed in line with the principles of the Helsinki Declaration and according to ethic board approval no. 236/12, University of Bonn. Patients underwent BM biopsy resulting in a diagnosis of primary myelofibrosis (PMF, *n* = 25), polycythemia vera (PV, *n* = 17), essential thrombocythemia (ET, *n* = 12), MDS (*n* = 16), MPN/MDS overlap syndromes (*n* = 16), AML (*n* = 13), or for staging purpose without showing pathology (normal, *n* = 23).

### 2.2. Immunohistochemistry and Scoring

Standard morphology was assessed using hematoxilin eosin (H&E), chloracetic acid (ASD) and Gordon silver-stained slides. Myelofibrosis grading was performed according to the WHO criteria for MPN [20]. NRP2 immunohistochemistry (IHC) on standard paraffin sections (2–3 μm) was performed manually as previously described with the addition of avidin/biotin blocking as described by the manufacturer (Avidin/Biotin Blocking Kit, Vector Laboratories, Burlingham, CA, USA) [21]. For other staining, epitope retrieval was performed at pH6 (Medak PMB-1-250; 20 min at 99 °C) and sections were developed using DAB IHC detection system on a semi-automatic immunohistochemistry stainer (Autostainer 480S; Medac, Wedel, Germany) [22]. For double staining, slides were first stained for NRP2, followed by staining for NCAM1/CD56 using HistoGreen HRP Substrate (Histoprime, Eching, Germany). The following primary antibodies were used: antibodies directed against NRP2 (aNRP2-36v2, 15 µg/mL, aTyr Pharma, San Diego, CA, USA), CD56/NCAM1 (clone RCD56; 1:100; Zytomed Systems, Berlin, Germany), SATB2 (clone EP281, 1:100, Cell Marque, Rockling, CA, USA). Photomicrographs were taken with a BX51 microscope (Olympus, Hamburg, Germany) and a Zeiss AxioCam MRc5 camera using the Axiovision software (Axiovision Viewer version 4.9, Carl Zeiss, Oberkochen, Germany). The amount of NCAM1 and NRP2 staining was scored using a semiquantitative scoring system as follows: No staining (0), endosteal linage cells positive (1), endosteal linage cells and osteoblasts positive (2), endosteal linage cells and osteoblasts positive as well as positive spindle cell proliferates (3), spindle cell proliferates positive aligned in groups (4). 

### 2.3. Cell Culture and In Vitro Osteoblast Differentiation

Murine pre-osteoblast cell line MC3T3-E1 subclone 4 was purchased from ATCC (Manassas, VA, USA) and maintained in ascorbic acid-free MEMα medium) supplemented with 10% (*v*/*v*) fetal bovine serum and 1% (*v*/*v*) penicillin/streptomycin (all Thermo Fisher Scientific, Paisley, UK). For osteoblast differentiation, 2 × 10^5^ (or) 4 × 10^4^ MC3T3-E1 wild type (WT) or Nrp2-KO cells were seeded in 6-well (or) 24-well plates and cultured in complete MEMα medium supplemented with 50 µg/mL ascorbic acid 2-phoshate, 10 mM β-glycerophosphate and 0.1 µM dexamethasone (Merck, Darmstadt, Germany) for 1, 3, 7, 14 and 21 days. For 3’ RNAseq analysis, medium without dexamethasone was used. Mineralization was visualized by Alizarin Red S staining (Merck, Darmstadt, Germany) and quantified after extraction with 10% (*w*/*v*) cetylpyridinium chloride (Merck,) as previously described [23]. Western Blot analysis of Nrp2 (D39A5; 1:1000; Cell Signaling Technology, Danvers, MA, USA) and Ncam1/CD56 (clone RCD56; 1:1000; Zytomed Systems) was performed as described [24].

### 2.4. scRNASeq Analysis

scRNAseq datasets were sourced from Leimkuehler et al. [8], Baccin et al. [25] and Li et al. [26]. For Li et al. [26], the raw counts were downloaded from GEO as outlined in the publication and the data were analyzed with SCANPY python package version 1.3.9 [27] using the Python script provided in the publication [26]. Data visualization of all scRNAseq datasets was performed using DimPlot, FeaturePlot, DotPlot, VlnPlot and DoHeatmap functions from Seurat R package version 5.0.0 [28,29]. Comparative statistical analysis for cell populations was performed using FindMarkers function from Seurat R package. Overrepresentation of NRP2 and NCAM1 positive cells was computed using Fisher.test function in R and Bonferroni correction was used to correct the corresponding *p* values. The analysis code is available at: https://github.com/anshupas/VosbeckEtal2024 (accessed on 24 February 2024).

### 2.5. CRISPR/Cas9-Mediated Gene Editing of MC3T3-E1 Cells

Nrp2-deficient MC3T3-E1 cells were generated by nucleofection of CRISPR/Cas9 ribonucleoprotein complexes (IDT, Leuven, Belgium) using a 4D nucleofector system (Lonza, Cologne, Germany) and the Amaxa SE Cell Line 4D-Nucleofector X Kit (catalog-no. V4XC1032, Lonza) according to the manufacturer’s instructions. Resulting clones were checked by genotyping, mRNA and protein expression for the absence of Nrp2 (Supplementary Methods and Data).

### 2.6. RNAseq Analysis of MC3T3-E1 Clones

As described above, 1 × 10^4^ MC3T3-E1 cells were seeded in 6-well plates and grown to confluence before starting osteoblast differentiation. RNA was extracted on day 0 (untreated) and day 6 using the RNeasy Plus Mini Kit (Qiagen, Hilden, Germany). QuantSeq 3′ mRNA-Seq Library Prep Kit (Lexogen, Vienna, Austria) was used for enrichment; sequencing was performed on a NovaSeq 6000 (Illumina, San Diego, CA, USA) device with read length of 1 × 100 bp. Data were provided in FastQ format and further analyzed by the Core Unit for Bioinformatics Data Analysis (CUBA). To pre-process 3’ transcript RNASeq data, nf-core/-rnaseq pipeline [30] was used in its default setup with GRCm38 mouse genome reference. Statistical analysis was performed in the R environment (R Core Team 2019; https://www.r-project.org/ (accessed on 20 April 2022)) with the Bioconductor package DESeq2 [31,32]. Benjamini–Hochberg method was used to calculate multiple testing adjusted *p* values. Data visualization was generated upon TMM normalized data [33] using R packages ggplot2 [34] and ComplexHeatmap [35], respectively. 

### 2.7. Cell Morphology

For morphological analysis, WT and Nrp2-KO MC3T3-E1 cells were grown to 80% confluency under standard culturing conditions and fixed with 4% paraformaldehyde (pH 7) in PBS (Thermo Fisher Scientific, Karlsruhe, Germany) for 5 min. After washing, cells were permeabilized with 0.25% Triton X-100 (Sigma–Aldrich, Darmstadt, Germany) for 5 min, stained using anti-actin (10 µg/mL; Abcam, Cambridge, UK) for 10 min and counterstained using 4’,6-Diamidino-2-phenylindole (DAPI), as described [23].

### 2.8. Cell Proliferation

Proliferation of MC3T3-E1 WT and Nrp2-KO cells was measured using an MTT assay according to the manufacturer’s instructions (Boster Biological Technology Co., Ltd., Pleasanton, CA, USA). Cells were cultured at a density of 3 × 10^3^ cells/well in a 96-well plate as stated above for 21 days. 

### 2.9. Analysis of Nrp2 Knockout Mouse Femurs

*Nrp2*-*flox* mice (JAX strain # 006697; *Nrp2 tm1.1Mom*/*MomJ,* Jackson Laboratories), further on named *Nrp2^fl^*^/*fl*^, were used as described [19]. *Nrp2* knockout mice were derived from *Nrp2-flox* by crossing with *CMV-Cre* mice (C57BL/6 background) and correspond in genotype to *Nrp2*-*Delta* (JAX strain # 006700; *Nrp2 tm1.2Mom*/*MomJ*), renamed as *Nrp2^−^*^/*−*^ in this manuscript. All mice used were fully backcrossed to C57BL/6J. Animals were kept in the animal facility of the University Hospital Bonn according to FELASA standards. Two animals of genotypes *Nrp2^fl^*^/*+*^ (control) and *Nrp2^−^*^/*−*^ were sacrificed by cervical dislocation for organ removal and analysis, respectively.

## 3. Results

### 3.1. NRP2 and NCAM1 Expression Is Increased in Myeloid Neoplasms Associated with Myelofibrosis In Situ

**Figure 1:**
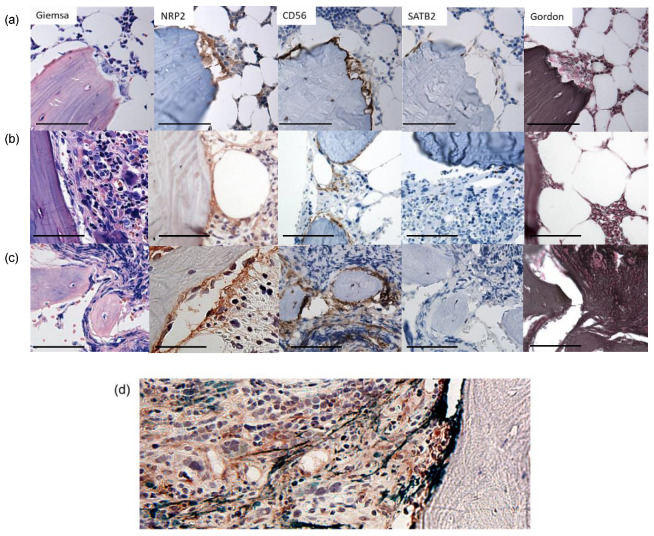

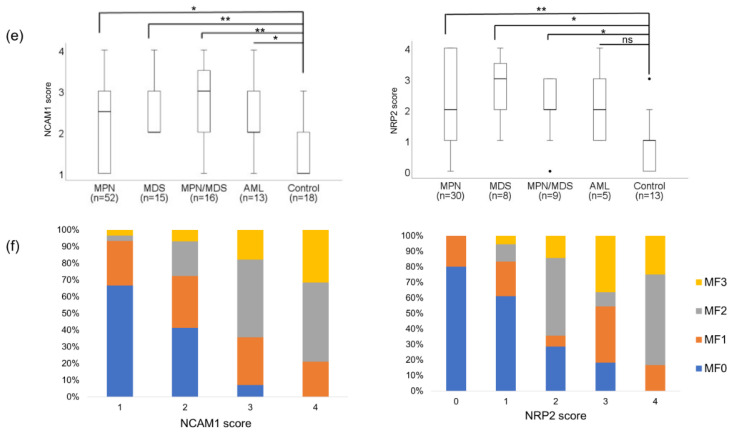
NRP2 and NCAM1 positive cells are increased in myelofibrosis in situ. (**a**–**d**) Immunohistochemical (IHC) stains as indicated in figure. (**a**) Normal bone marrow undergoing osteo repair showing osteoclasts adjacent to bone. (**b**) PMF with low fibrosis (MF-1), no osteosclerosis (OS-0). (**c**) PMF with end-stage fibrosis (MF-3, OS-3) showing irregular, thickened osteoid/osteosclerosis. NCAM1 and NRP2 IHC positive cells are found in layers around bone and diffuse within the intramedullary cavity. (**d**) PMF MF-3, double immunostaining NRP2 (brown) and NCAM1 (green), double stained cells can be found in the endosteal niche as well as diffuse in the bone marrow stroma. (**e**) Box plots displaying NCAM1 and NRP2 scores per disease entity including MPN, MDS, MPN/MDS, AML, *p* as indicated in figure, chi square. Outliers are indicated as black dots (.) (**f**) Stacked bar graph comparing MF grade with NCAM1 and NRP2 IHC Scores. Statistical significance was defined as * *p* < 0.05, ** *p* < 0.01, ns: not significant (*p* > 0.05) (photomicrograph, Olympus BX41, Axiovision), scale bar represents 50 µm, original magnification ×400.

To localize and identify NRP2- and NCAM1-expressing cells under non-pathologic and fibrotic conditions, we performed immunostaining on BM biopsies. Patient characteristics are listed in Table 1. In normal BM, NRP2 co-localized with NCAM1 in sparse endosteal lining cells (Figure 1a). While NCAM1 encircled bony spicules separating bone from normal BM, NRP2 was found more sparsely, but also within the endosteal niche (Figure 1a). In areas of bone remodeling, osteoclasts and osteoblasts expressed NRP2 simultaneously (Figure 1a). Cuboidal-shaped osteoblasts, but not quiescent osteocytes, also expressed NCAM1 and SATB1 (Figure 1a–c).

During early myelofibrosis in PMF, NRP2 and NCAM1 expression was increased in the endosteal niche (Figure 1b). Advanced myelofibrosis was characterized by stromal spindle-shaped cells expressing both NRP2 and NCAM1 in aggregates and diffusely within the bone marrow space resulting in progressive osteosclerosis with anastomosing spicules (Figure 1c,d). 

NCAM1 and NRP2 expression was increased in MPN, MDS and MPN/MDS compared to normal BM (Figure 1e). Statistical analysis revealed increased and aberrant NCAM1 (*p* < 0.001) and NRP2 expression (*p* = 0.015) with increased grades of fibrosis (Figure 1f). Furthermore, NCAM1 and NRP2 expression scores correlated with each other (*p* < 0.001). AML cases showed a trend of correlation of NCAM1 with fibrosis, but case numbers were too low for statistical analysis.

### 3.2. Phenotypic Identification of NRP2 and NCAM1 Co-Expressing MSC in Normal BM

**Figure 2:**
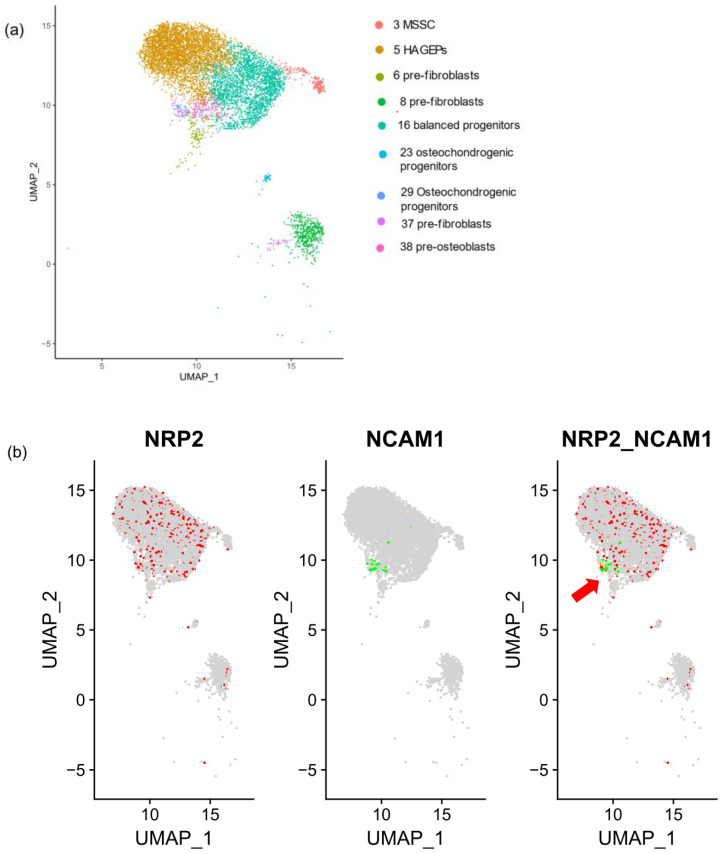
NRP2 expression in human BM multipotent MSC by scRNA seq analysis, non-pathological. ScRNA seq analysis (dataset Li et al., 2023 [28]). (**a**) UMAP plots showing stromal clusters of human bone marrow scRNAseq, subsets and genes as indicated. (**b**) NRP2 (red) and NCAM1 (green) are co-expressed (yellow) in osteochondrogenic progenitor stromal cells annotated as cluster 29 by Li et al. (*p* = 0.0016, Fisher’s test, Bonferroni corrected, indicated by red arrow), while NRP2 is expressed more broadly within the CXCL12^+^ MSC cluster including balanced progenitors and pre-fibrogenic cell types.

To identify NRP2-expressing MSC subclusters, we analyzed NRP2 and NCAM1 mRNA expression using scRNA MSC-enriched human BM data [26]. Li et al. define nine stromal cell clusters including adipogenic, pre-fibroblast, S100A4/S100A6-expressing pre-fibroblasts, balanced osteo/adipo-progenitor, osteo/chondrogenic progenitors, pre-fibroblasts and pre-osteoblasts [26] (Figure 2a). We found NRP2 to be expressed in multipotent MSC, including adipogenic and osteogenic MSC [26]. Co-expression of NRP2 and NCAM1 was observed in a small fraction of stromal cells annotated as osteochondrogenic (“cluster 29”) cells [26] (*p* = 0.0016, Figure 2b). 

**Figure 3:**
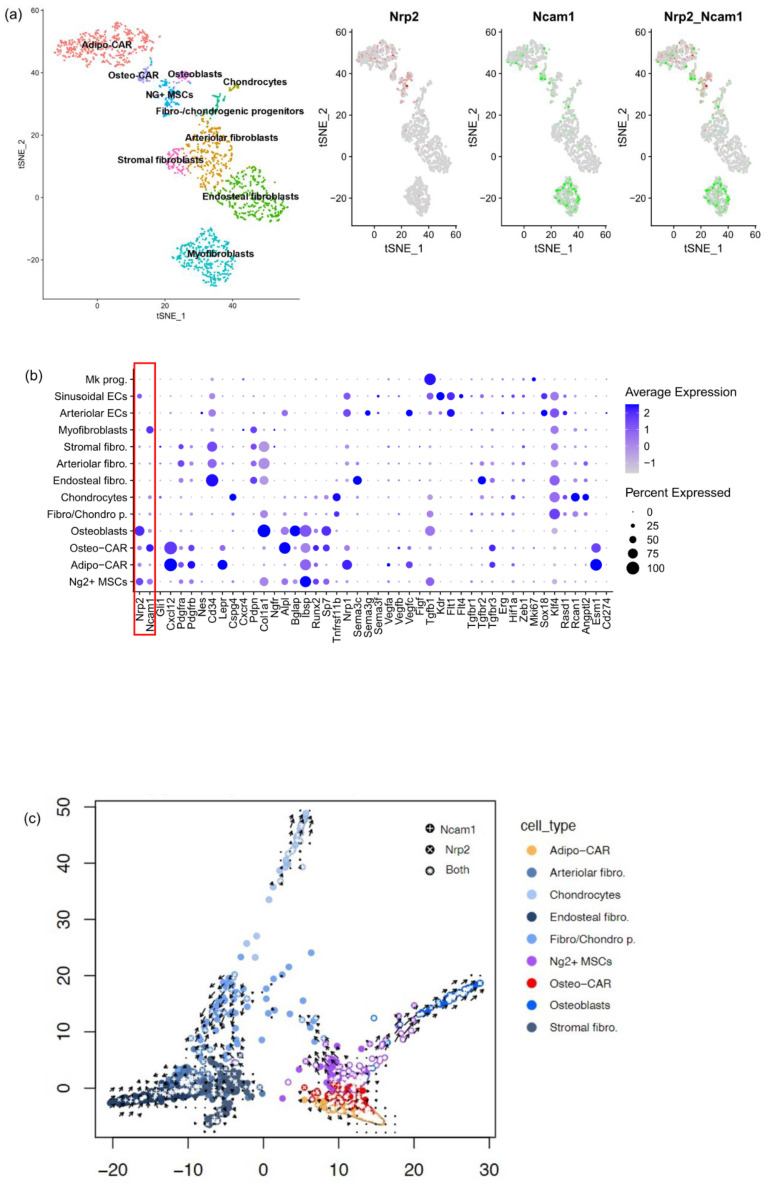
*Nrp2* expression in MSCs and osteolineage cells by scRNA seq analysis, mouse bone marrow under non-pathological condition. (**a**) UMAP plot displaying *Nrp2* and *Ncam1* co-expression in mouse BM MSC compartment. *Nrp2* is expressed in Adipo-CAR, Osteo-CAR, Ng2^+^ MSC and osteoblasts, while *Ncam1* is expressed in Adipo-CAR, Osteo-Car, Ng2^+^ MSC and myofibroblasts. *Nrp2* and Ncam1 double-expressing cells are overrepresented in Ng2^+^ MSC (*p* < 0.05), arteriolar fibroblasts (*p* < 0.05) and endosteal fibroblasts (*p* < 0.005, Fisher’s test, Bonferroni corrected). (**b**) Dot plot ligand/receptor analysis revealed *Sema3c* and *Tgfbr2* co-expressed in endosteal fibroblasts, while negative for *Nrp2*. Nearby osteoblasts co-express *Nrp2* and *Ncam1 (*red frame) but are negative for Vegf signaling receptors (*Flt1, Kdr, Flt4*). *Vegf*-receptor expression is found exclusively on endothelial cells with selective *Flt4* expression specific in sinusoidal endothelial cells. (**c**) Velocity plot showing *Nrp2* and *Ncam1* double positive cells (*) in the MSC trajectory. Arrows indicate the trajectories of cell cluster differentiation. Color coding and analysis as in Baccin et al. 2019 [25]. (+) indicates *Ncam1* positive cells, (×) indicates *Nrp2* positive cells.

We found similar expression patterns in a murine BM MSC [25]. Here, Cxcl12-abundant reticular (CAR) populations were previously annotated as Adipo-CAR and Osteo-CAR [25] (Figure 3a–c and Figure S1). A fraction of both CAR subclusters and osteoblasts expressed both Nrp2 and Ncam1 (Figure 3a–c). In addition, sole Nrp2 expression occurred in osteoblasts, Adipo-CAR, Osteo-CAR, Ng2^+^ MSCs and sinusoidal endothelial cells (EC) (Figure 3b). Furthermore, Nrp2 and Ncam1 double expression was observed in sparse arteriolar fibroblasts (*p* < 0.05), endosteal fibroblasts (*p* < 0.005) and Ng2^+^ MSC (*p* < 0.05). Ncam1 was expressed in myofibroblasts, negative for Nrp2.

Re-analysis of previously published velocity plot analysis [25] allowed an overlay of Nrp2- and Ncam1-expressing cells (Figure 3c). We found enrichment of Nrp2 positive cells ranging from low in Adipo-CAR, Osteo-CAR, Ng2^+^ MSC to high in osteoblasts (Figure 3b), following the osteogenic trajectory (Figure 3c). Ncam1 and Nrp2 expression were identified within the fibrogenic trajectory, while Ncam1 was more prominently expressed along the chondrogenic trajectory (Figure 3c). 

Nrp2 function depends on its interaction with signaling receptor/ligand pairs, such as Vegfs and Vegf receptors. Only EC have signaling Vegf receptor expression: Vegfr1/Flt1, Vegfr2/Kdr and Vegfr3/Flt4 are all expressed in sinusoidal EC, while Vegfr1/Flt1 was the only Vegfr in arteriolar EC (Figure 3b). On the other hand, the ligand Vegfc was selectively expressed in Adipo-CAR, Osteo-CAR and arteriolar EC (Figure 3b), suggesting Vegfc-Nrp2 binding in sinusoidal EC, which is consistent with previous reports [36].

Transforming growth factor (TGFβ) signaling is pivotal for fibrogenesis and osteogenesis, with NRP2 being involved in binding of TGFB1 and its receptors [9]. Upon re-analyzing murine BM [25] scRNAseq data, we found Tgfb1 predominantly expressed in megakaryocyte precursors (Figure 3b) and slightly less in osteoblasts, Ng2^+^ MSC and sinusoidal EC, coinciding with elevated Nrp2 expression. Conversely, we found the signaling receptor Tgfbr2 to be expressed on spatially adjacent endosteal fibroblasts, stressing Tgfβ signaling along the fibrogenic trajectory. Expression of the proteoglycan Tgfbr3 was confined to Adipo-CAR and Osteo-CAR cells (Figure 3b,c), suggesting inhibition of Tgfβ signaling in these cells. While Vegf signaling receptors specific for Vegfc were only expressed in sinusoidal EC and Adipo-CAR cells in murine BM, our ligand/receptor analysis suggests Tgfβ signaling to play a Nrp2-dependent role in fibrogenesis. 

### 3.3. NRP2 and Ligand Expression in Murine Models of Myelofibrosis

**Figure 4:**
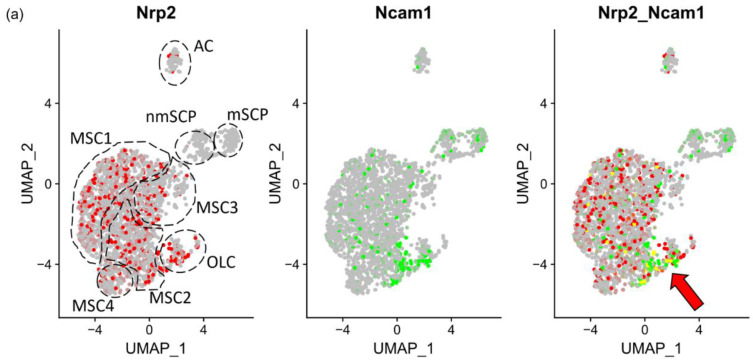

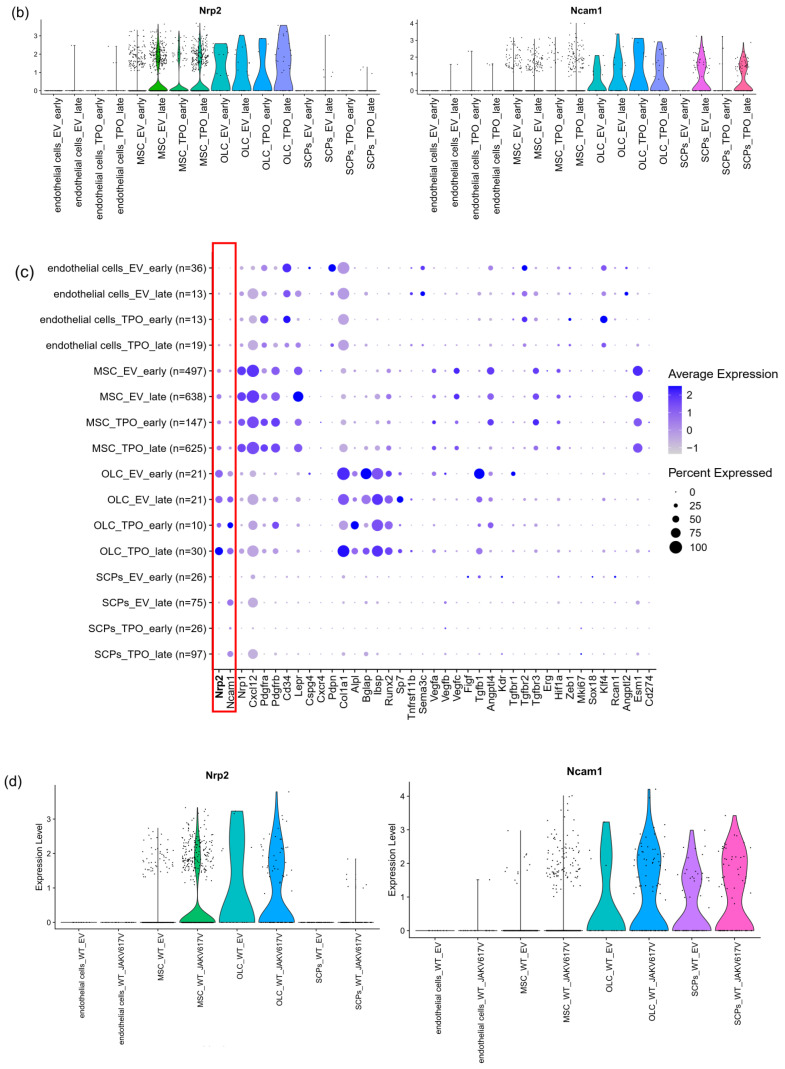

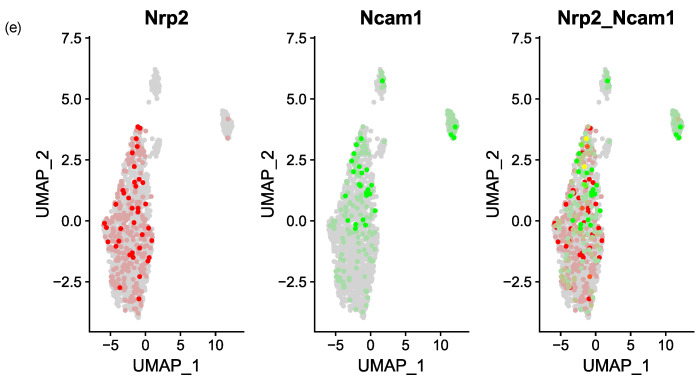
Increased number of Nrp2 expressing osteolineage and fibrosis promoting MSCs identifies Nrp2 as a druggable target in murine myelofibrosis. (**a**) UMAP plots of scRNAseq reanalysis (Leimkühler et al., 2020 [8]) with Nrp2 (red), Ncam1 (green) or co-expression (yellow, red arrow) of both genes within MSC subclusters in the ThPO lentiviral murine myelofibrosis model. Co-expressing cells were primarily found in MSC2 and OLC clusters. Cells expressing neither Nrp2 nor Ncam1 are labelled in grey. (**b**) Violin plots of the ThPO murine myelofibrosis model. Nrp2 and Ncam1 expression within endothelial cells, MSC, OLC and SCPs comparing early and late fibrotic stage. (**c**) Dot plot including Nrp2 ligands and receptors in the ThPO murine myelofibrosis model. Nrp2 tended to be upregulated in OLC early vs. late ThPO (Wilcoxon test, Bonferroni corrected). Expression level is encoded by color, enrichment in subclusters by circle size. Red frame indicates expression of Nrp2 and Ncam1. (**d**) Violin plots of MSC clusters in the JAK2*^V617F^* knockin myelofibrosis model. Nrp2 is upregulated in MSC and osteolineage cells sparing Schwann cells, the latter expressing Ncam1. (**e**) UMAP plots of Nrp2 (red), Ncam1 (green) or co-expression (yellow) of MSC subclusters in the JAK2*^V617F^* knockin mouse model. For cluster assignment see Leimkühler et al. [8]. Cells expressing neither Nrp2 nor Ncam1 are labelled in grey.

In order to gain more information regarding Nrp2 involvement in myelofibrosis, we examined the expression of the genes introduced above in established murine models of MPN-associated fibrosis [8]. In the ThPO lentivirus-induced model [8], Leimkühler et al. annotate osteolineage cells (OLC), MSC, EC and Schwann cell progenitors (SCP). Here, we found Nrp2 and Ncam1 double-expressing cells within MSC2 (osteogenic MSC), MSC3 (transitioning MSC) and OLC (Figure 4a–c). Col1a1 and osteogenic differentiation markers Alpl and Runx2 were expressed in OLC, coinciding with the expression of Nrp2 and Ncam1 (Figure 4c). Together, these findings suggest that Nrp2 and Ncam1 double-expressing cells represent rare MSC able to differentiate toward the fibrotic and the osteogenic trajectory driving myelofibrosis and osteosclerosis. No Nrp2 expression was observed in Ncam1 positive SCP, neither under normal nor under fibrotic conditions. Expression levels in endothelial cells could not be accurately assessed due to low cell counts, but Vegfc was increased predominantly in MSC during late-stage fibrosis. Tgfb1 was overrepresented in OLC during fibrosis compared to other stromal cell types. 

The JAK2*^V617F^* transgenic mouse model [8] showed similar results as the ThPO lentiviral model. Both Nrp2 and Ncam1 were overrepresented in fibrotic OLC while *Nrp2* expression was also upregulated in fibrotic MSC (Figure 4d). Nrp2 and Ncam1 double positive cells in the JAK2*^V617F^* mouse model were not restricted to a single MSC subcluster as defined by Leimkühler et al. [8]. (Nrp2_Ncam1, Figure 4e).

### 3.4. Nrp2 Is Associated with Increased Osteogenic Differentiation In Vitro

**Figure 5:**
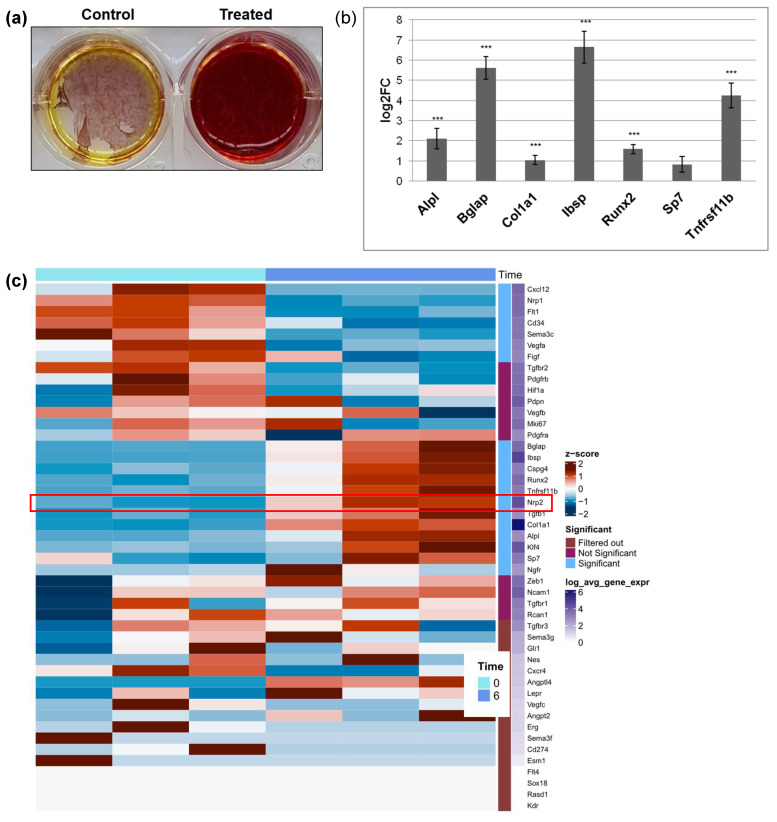
In vitro upregulation of Nrp2 during osteogenic differentiation of MC3T3-E1 cells. (**a**) Osteogenic differentiation and mineralization shown by alizarin red staining, day 21. (**b**) Bar graph showing osteogenic marker gene expression after treatment of pre-osteoblasts with ascorbic acid and β-glycerophosphate, RNAseq, day 6. (**c**) Heat map of RNAseq clustering showing gene expression in three independent experiment samples on day 0 and day 6 of differentiation. Statistical significance was defined as *** *p* < 0.001.

To validate these findings in a functional setting, next, we used an in vitro model to study the function of Nrp2 (and Ncam1) during osteoblast differentiation. MC3T3-E1 cells [18] are pre-osteoblasts that can differentiate toward mineralizing osteoblasts in vitro (Figure 5). During osteogenic differentiation, these cells downregulate Cxcl12 and increasingly calcify (Figure 5a). Already at d6, MC3T3-E1 expressed osteogenic markers such as Bglap, Ibsp, Col1a1, Runx2, Alpl and Tnfrsf11b/osteoprotegerin (Figure 5b), resembling maturing pre-osteoblasts in vivo. Like in osteogenic trajectories (Figure 3c), *Ncam1* was not significantly altered, while Nrp2 and Tgfb1 were upregulated upon osteogenic differentiation (Figure 5c, red frame). During this process, Nrp1, Cxcl12, Vegfr1/Flt1, CD34, Sema3c, Vegfa and Vegfd/Figf were all downmodulated (Figure 5c). These findings further indicated a role of Nrp2 in osteogenesis and prompted us to generate *Nrp2* knockout cells for further analysis. 

### 3.5. CRISPR/Cas9-Mediated Nrp2 Ablation in MC3T3-E1 Cells Generates an Osteogenic Differentiation Defect In Vitro

**Figure 6:**
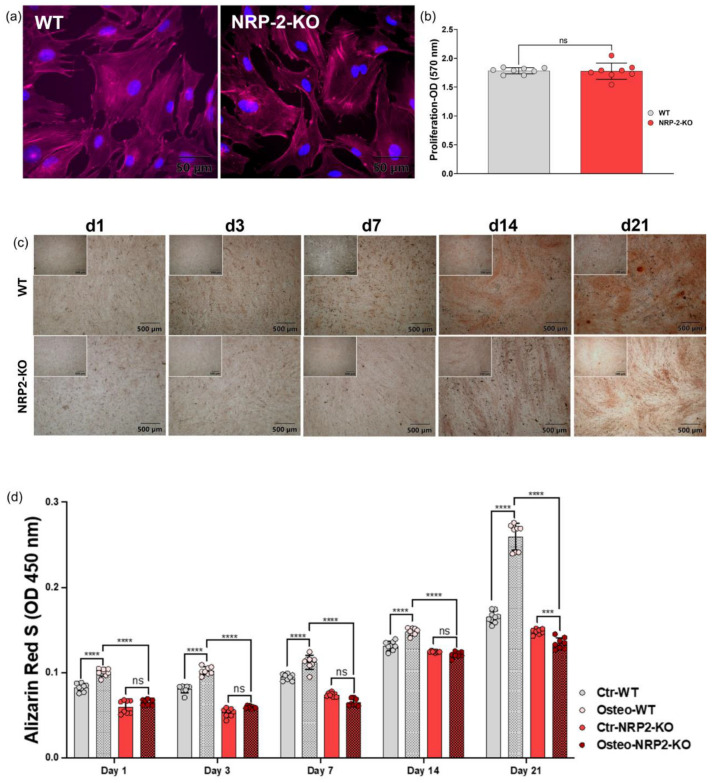

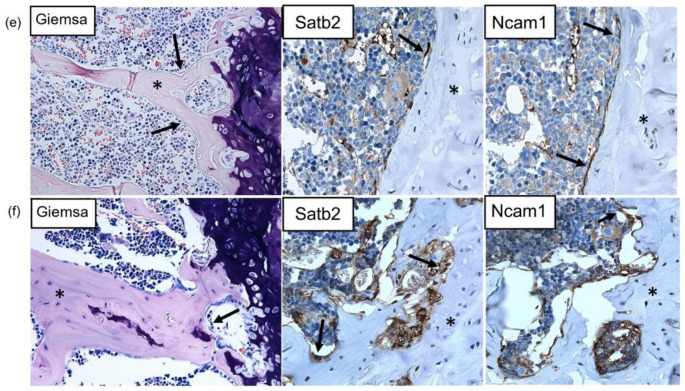
*Nrp2* ablation modulates osteogenic differentiation in vitro and in vivo. (**a**) MC3T3-E1 WT and MC3T3-E1 *Nrp2*-KO cells (Supplemental Methods and Data) are similar in morphology: anti-cytoskeleton-actin (red) and nuclear morphology (DAPI, blue). (**b**) MC3T3-E1 WT and *Nrp2*-KO cells show similar growth by MTT assay (absorbance, 570 nm) at day 21. (**c**) MC3T3-E1 WT and *Nrp2*-KO cells were induced to mineralize for up to 21 days. Culturing medium without any supplements was used as control (left upper corner). Photomicrographs demonstrate mineralization as confirmed by alizarin red S staining on indicated days in culture. (**d**) Quantification of alizarin red staining by OD measurement, comparing osteogenic-induced (Osteo-WT, Osteo-*Nrp2*-KO) and non-induced cells (Ctr-WT, Ctr-*Nrp2*-KO). Statistical significance in (**b**,**d**) was defined as *** *p* < 0.001, ****, *p* < 0.0001, ns: not significant. (**e**) Histology, femoral bone of WT and *Nrp2^−^*^/*−*^ mice, age 6–10 weeks (Giemsa, 400×, Olympus BX41), *Nrp2*^−/−^ bony spicules are narrow (*) and lined by a thin layer of flattened osteolineage cells (Satb2, Ncam1), rather than mature cuboidal osteoblasts observed near metaphysis of control mice; arrows showing osteoblasts (->). (**f**) Control mice (*Nrp2^fl^*^/*+*^) (Giemsa, 400×, Olympus BX41) showing mature cuboidal osteoblast morphology (Giemsa).

To assess the functional role of Nrp2 in vitro, we generated MC3T3-E1 *Nrp2* knockout cell lines by CRISPR/Cas9-mediated deletion of exon 3 in the murine *Nrp2* locus (Supplementary Methods and Data). Both WT and *Nrp2* KO cells showed a similar morphology (Figure 6a) after actin labeling with no significant difference in their growth behavior up to 21 days in culture (Figure 6b). However, when induced toward the osteogenic lineage, MC3T3-E1 WT cells showed a 2-fold excess in mineralization compared with MC3T3-E1 *Nrp2* KO cells (Figure 6c,d) on day 21. Western blot analysis confirmed the upregulation of Nrp2 protein during osteogenic maturation in the presence of unchanged Ncam1 protein expression (Figure S2). 

### 3.6. Osteopenia by Nrp2 Loss In Vivo Is Accompanied by Altered Osteoblast Morphology

To assess the functional impairment in osteolineage cells in vivo, we evaluated alterations in osteoblast morphology in *Nrp2*^−/−^ adult mouse femurs. Histologically, *Nrp2^−^*^/*−*^ osteoblasts near the femoral metaphysis appeared flattened compared to normal cuboidal morphology, surrounding narrower bony spicules (trabeculae), as demonstrated in Giemsa-stained sections (Figure 6e,f). Expression of osteoblast markers Satb2 and Ncam1 was unaltered in both *Nrp2*^−/−^ and *Nrp2*^fl/+^ control mice (Figure 6e,f), indicating that osteoblasts were not replaced by other cell types, but instead retained a more immature morphology accompanied by impairment in mineralization. This is concordant with earlier in vivo results in *Nrp2*^−/−^ mice [37,38] that show osteopenia with thinner bone spicules and a decrease in osteoid-producing osteoblasts in femoral long bones. The above in vitro and in vivo results suggest that NRP2 is involved in osteolineage maturation.

## 4. Discussion

Here we show that NRP2 is involved in fibrogenic and osteogenic MSC trajectories and is elevated in MPN, MDS and MPN/MDS overlap syndromes. In fact, NCAM1 and NRP2 protein expression correlated with the severity of myelofibrosis in our clinical cohort. 

Both the identity of NRP2 positive mesenchymal stromal cells (Figure 3 and Figure 4) as well as functional data in NRP2 knockout pre-osteoblasts (Figure 6) point toward a function of NRP2 during osteogenic matrix formation. Further studies are needed to investigate whether NRP2 inhibition results in less fibrosis in vitro and in vivo and how NRP2 ligands orchestrate different differentiation pathways during myelofibrosis. Furthermore, it would be interesting to unravel the exact mechanisms of how NRP2 increases osteogenic differentiation on a molecular level in osteoblasts. Further investigation is needed using in vitro and in vivo NRP2-modulating agents, similar as in other types of fibrotic disease [39].

Osteolineage cells and derived endosteal osteoblasts support malignant hematopoietic clones to promote myeloproliferation-induced fibrosis [40]. Human BM scRNAseq [26] analysis revealed NRP2 expression in multipotent MSC, while NRP2/NCAM1 co-expressing cells were only identified in a subset of osteochondrogenic progenitor cells (Figure 2). Analysis of the murine BM scRNAseq dataset [25] refined these results as Nrp2 and Ncam1 were co-expressed within immature osteogenic and fibrogenic trajectories (Figure 3). Consistent with and extending our in situ findings in clinical patients, Nrp2 was also expressed in profibrotic, osteogenic and inflammatory MSC and osteolineage cells in murine myelofibrosis models [8] by scRNAseq analysis (Figure 4), supporting its possible role as a druggable target in MPN. Lack of NRP2 expression in Schwann cells in this dataset is particularly interesting, as neuropathy has been reported to worsen myelofibrosis in MPN [41]. 

We identified Adipo-CAR and arteriolar EC as major sources of Vegfc expression in murine BM (Figure 3) which can bind to Vegfr positive sinusoidal EC in an *Nrp2*-dependent manner. It is known that following myeloablation, Vegfc and Vegfr3/Flt4 are necessary for endothelial regeneration of sinusoidal EC and reconstitution of hematopoiesis [36]. While NRP1 is expressed in both arteriolar and sinusoidal EC (Figure 3b), NRP2 is restricted to sinusoidal EC. This could explain that in contrast to NRP1 inhibition [42], no significant hematologic impairment has been observed in phase III clinical trials targeting NRP2 in pulmonary fibrosis (NCT03824392, NCT04412668) [43]. Furthermore, hematopoiesis appears unaffected in osteopenic *Nrp2*^−/−^ mice [37], supporting this notion. 

Apart from binding ligands of the VEGFR family, NRP2 serves as a co-receptor for TGFβ1. In PMF, tight clusters of TGFβ1 positive neoplastic megakaryocytes found immediately adjacent to bone distinguish PMF from other types of MPN (Figure 1), suggesting TGFβ1 signaling to NRP2-expressing osteolineage and profibrotic MSC within the endosteal niche. This is supported by observations that MSC in PMF have greater profibrotic and osteogenic potential and a TGFB1-signaling signature [44]. In normal murine BM, we found sole Tgfb1 expression in the absence of Nrp2 confined to megakaryocyte progenitors (Figure 3b). In contrast, Nrp2 was co-expressed with Tgfb1 in Ng2^+^ MSC and osteoblasts, suggesting an autocrine function in these cells. It appears that also endosteal fibroblasts are potential target cells for aberrant fibrosis, as they expressed Tgfbr2 as a signaling component within this axis (Figure 3b). 

TGFβ is a master regulator in multiple MSC during myelofibrosis [8] and other organ type fibrosis [45]. Given its function in TGFβ signaling [46,47], NRP2 emerges as an interesting novel druggable target to ameliorate myelofibrosis and osteosclerosis simultaneously in PMF patients. The findings above are in line with previous data on the role of NRP2 during osteogenesis [48], fibrogenesis and angiogenesis [37]. 

While the role of Ncam1 in osteogenic differentiation has been demonstrated previously [49,50], here we give an insight of a functional role of Nrp2 in profibrotic and osteogenic MSC trajectories (Figure 2 and Figure 3) in vivo and during osteogenic differentiation of MC3T3-E1 cells in vitro (Figure 5a,b). Osteogenic differentiation is accompanied by upregulation of Nrp2 and Tgfb1, while Vegfs and Vegfrs are downregulated (Figure 5c). Nrp2 ablation in these cells seems to impair osteogenic differentiation (Figure 6). These data are in line with in vivo results showing that Nrp2 deficiency leads to diminished bone mass and density [37]. In femoral long bones of *Nrp2*^−/−^ mice [19], we found sparse osteoblasts with an immature pre-osteoblast morphology (Figure 6e,f) suggesting dysfunctional maturation. Interestingly, Nrp2 expression in osteoblasts appears to be regulated by the osteogenic differentiation marker Runx2 [51]. Recently, Verlinden et al. showed that Nrp2- related effects on bone differentiation are compensated by high-dosage vitamin D_3_ treatment [38], indicating a modulating effect of Nrp2 in this process. While our study gives insights into the functional role of NRP2 in osteogenic differentiation, further investigation in other osteoblast cell lines would be helpful. 

While TGFβ inhibitors are in early clinical trials [52,53], some anti-fibrotic or VEGFR, TGFBR receptor-inhibiting drugs proved to be toxic [54]. Thus, novel treatments are needed, especially in MPN patients not responsive to standard therapy and not eligible for BM transplant. NRP2 is a clinically druggable target in pulmonary fibrosis [55]. Here, we evaluated NRP2 as a mesenchymal stromal cell target in myelofibrosis by using mouse and human bone marrow and scRNAseq data. Our data suggest that modulation of NRP2 in patients with myelofibrosis may be beneficial to counteract fibrosis and osteosclerosis and first studies show that NRP2 modulation results in low toxicity profiles [43]. In contrast to NCAM1, we found no significantly elevated NRP2 expression compared to non-pathological BM in the limited number of AML biopsies, warranting further study. Experimental data support the idea that osteogenic inhibition in MPN could be of therapeutic benefit in myeloid neoplasms that depend on an osteoblast-rich stroma, such as shown in murine models of CML [56] and MDS/AML [57,58]. Thus, other myeloid neoplasms could also respond to NRP2 modulation.

## 5. Limitations of the Study

Interpretation of the scRNA-seq data is limited and awaits future confirmation using other data sets, due to low cell counts in the mesenchymal cell clusters.

## 6. Conclusions

In summary, co-expression of NRP2 and NCAM1 is confined to very early osteogenic precursor cells and increases upon osteogenic and fibrogenic differentiation, rendering previously clinically approved NRP2 modulators [43] which is a promising therapeutic modality to target fibrosis and osteosclerosis in MPN, MDS and MPN/MDS overlap syndromes.

## Figures and Tables

**Table 1:** Patient cohort and clinico-pathological data relating to Figure 1.

Diagnosis	PMF	ET	PV	MDS	MPN/MDS/ CMML	AML	Control	Total
Number of Cases. N (%)	25 (20)	12 (10)	17 (14)	16 (13)	16 (13)	13 (11)	23 (19)	122 (100)
Median Age (range)	63.6 (21–85)	64.3 (31–81)	57.8 (41–79)	75.4 (52–90)	66.8 (42–84)	58 (36–73)	58 (31–79)	63.4
Gender								
Male	16	7	8	12	14	8	12	77
Female	9	5	9	4	2	5	11	46
MF grade (%)								
0	1 (2)	10 (20)	5 (10)	4 (8)	4 (8)	3 (6)	23 (46)	50 (100)
1	5 (18)	2 (7)	5 (18)	4 (14)	6 (21)	6 (21)	0 (0)	28 (100)
2	11 (35)	0 (0)	6 (19)	7 (23)	3 (10)	4 (13)	0 (0)	31 (100)
3	8 (62)	0 (0)	1 (8)	1 (8)	3 (23)	0 (0)	0 (0)	13 (100)
Mutation								
JAK2*^V617F^*	13	6	12	0	1	0	0	32
CALR	5	1	0	1	2	0	0	9
NPM1	0	0	0	1	2	1	0	4
MPL	3	1	0	0	0	0	0	4
SRSF2	3	0	0	0	1	1	0	5
Mean haemoglobin [g/dL] (range)	9.9 (4.2–15.5)	14.1 (9.1–16.4)	14.2 (8.4–19.8)	9.4 (6.3–13.9)	9.2 (7–11.8)	10.2 (6.7–13.4)	11 (8.5–15.4)	11.1 (4.2–19.8)
Mean platelet [10^3^/µL] (range)	294.5 (8–993)	787.2 (23–1143)	380.6 (31–969)	83.8 (16–205)	222.1 (10–1462)	59.6 (13–130)	252.1 (82–618)	297.1 (8–1462)
Mean WBC [10^3^/µL] (range)	13.8 (3.8–36.21)	12.1 (2.09–14.42)	18.5 (1.57–32.49)	2.8 (0.3–6.83)	15.5 (1.78–56.04)	26.4 (1.2–25.62)	8 (5.17–11.53)	13.9 (1.2–56.04)

## Data Availability

All data in this study are available from the corresponding author (I. G.) upon request. The analysis code is available at: https://github.com/anshupas/VosbeckEtal2024 (accessed on 24 February 2024).

## Supplementary Materials

The following supporting information can be downloaded at: https://www.mdpi.com/article/10.3390/cancers16101924/s1, Supplementary Methods and Data: CRISPR/Cas9-mediated generation of Nrp2-deficient MC3T3-E1 cells; Figure S1: Gene expression in MSCs normal mouse bone marrow. Figure S2: Protein expression of MC-3T3 E1 cells during osteogenic differentiation in the presence or absence of Nrp2.

## Abbreviations

AML	acute myeloid leukemia
BM	bone marrow
CAR	Cxcl12-abundant reticular
EC	endothelial cells
ET	essential thrombocythemia
IHC	immunohistochemistry
KO	knockout
MDS	myelodysplastic syndromes
MF	myelofibrosis
MPN	myeloproliferative neoplasm
MSC	mesenchymal stromal cells
NCAM1	neural cell adhesion molecule 1
NRP2	neuropilin 2
OLC	osteolineage cells
PMF	primary myelofibrosis
PV	polycythemia vera
SCP	Schwann cell progenitors
ScRNAseq	single cell RNA sequencing
TGFβ1	transforming growth factor β1
VEGFs	vascular endothelial growth factors
WT	wild type
