# Supplementary material for: Neuropilin2 in Mesenchymal Stromal Cells as a Potential Novel Therapeutic Target in Myelofibrosis
Source: Cancers (Basel). 2024 May 18;16(10):1924. doi: 10.3390/cancers16101924 (PMC11119673; doi:10.3390/cancers16101924)

Supplementary Materials:

Supplementary Figures:

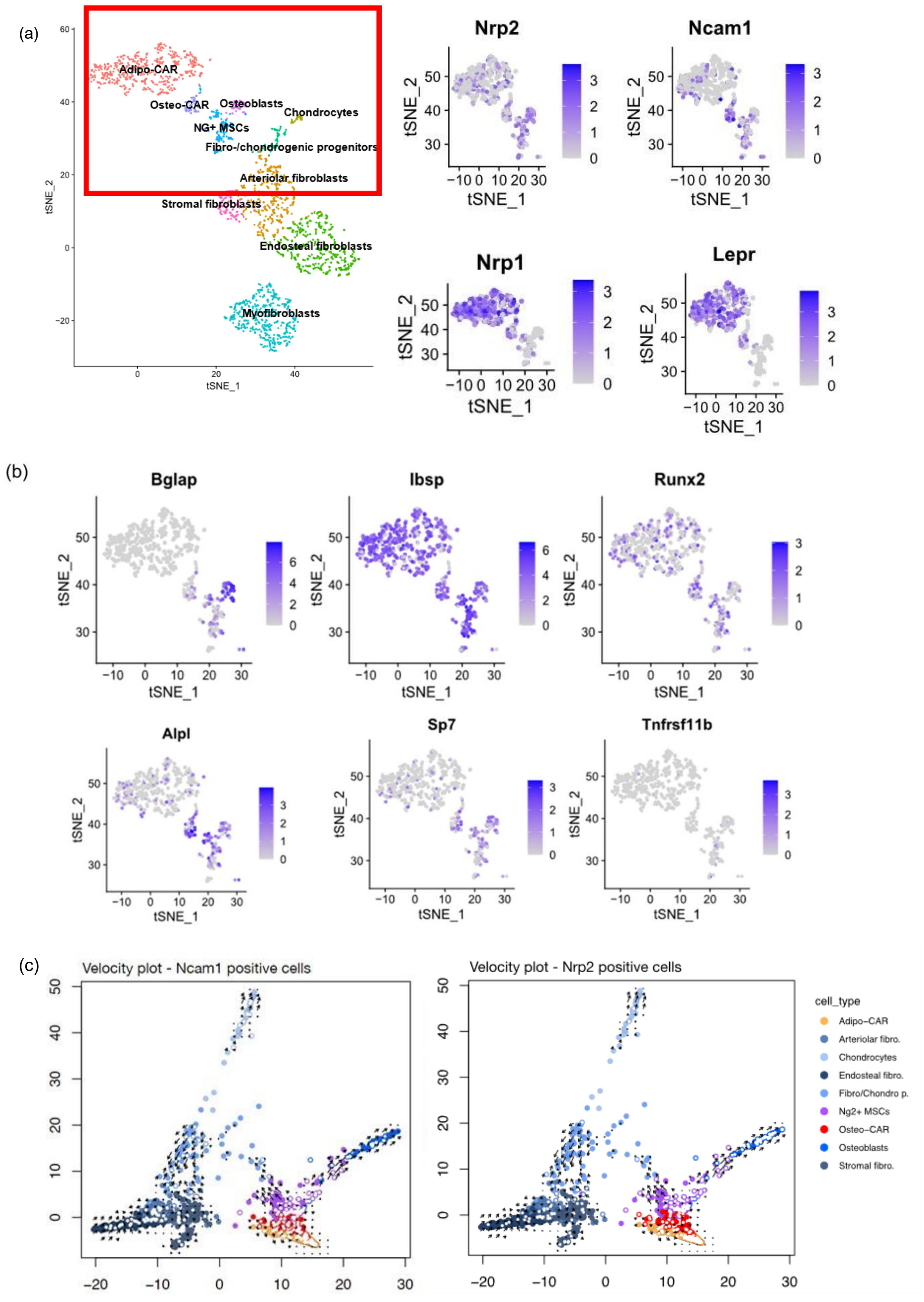

Figure S1. Gene expression in MSCs normal mouse bone marrow (A) Cell clusters gated for the following single gene analyses are marked by red frame. Nrp2 and Ncam1 expression shown in cell clusters including osteogenic lineage. Lepr is highly expressed in Adipo-CAR cells and shows a similar expression pattern to Nrp1. (B) Osteogenic markers (Bglap, suggesting an osteogenic potential in all of these cell clusters. (C) Velocity plots showing either Nrp2 (left) or Ncam1 (right) expression in the mesenchymal trajectories. Ibsp, Runx2, Apl, Sp7, Tnfrsf11b) are expressed in Adipo-/Osteo-CAR, Ng2+ MSC and osteoblasts, suggesting an osteogenic potential in all of these cell clusters. Grey dots label cells that do not express the gene of interest. (C) Velocity plots showing either Nrp2 (left) of Ncam1 (right) expression in the mesenchymal trajectories. Arrows indicate the trajectory of differentiation.

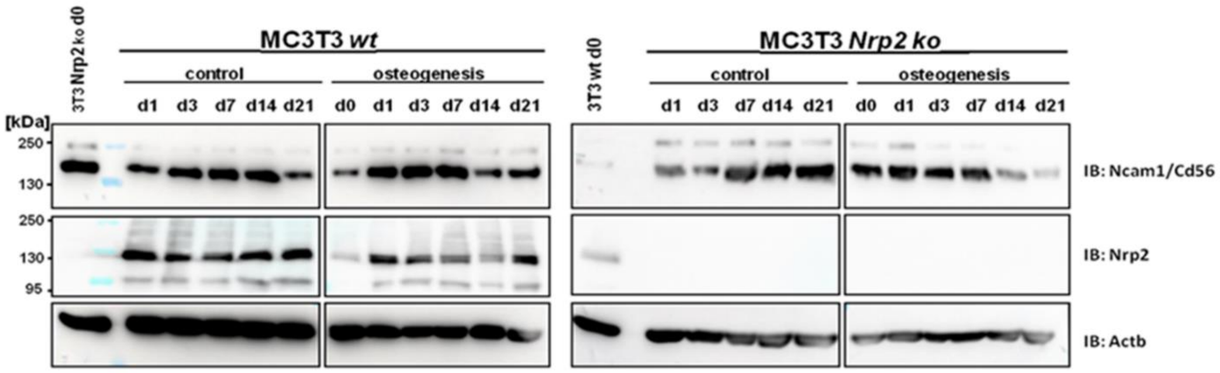

Figure S2. Protein expression of MC-3T3 E1 cells during osteogenic differentiation in the presence or absence of Nrp2. Western blot (see methods) showing knockout of Nrp2 (IB. Nrp2) in MC3T3 *Nrp2-KO* (right panel) versus MC3T3 wt (left panel) as compared to persistent expression of Ncam1 protein (IB: Ncam1) in both cell lines. Cell lines were subjected to either osteogenic differentiation conditions (osteogenesis) or control conditions (control) and analyzed at day 1 (d1), day 3 (d3), day7 (d7), day 14 (d14) and day 21 (d21).

Supplemental Methods and Data:

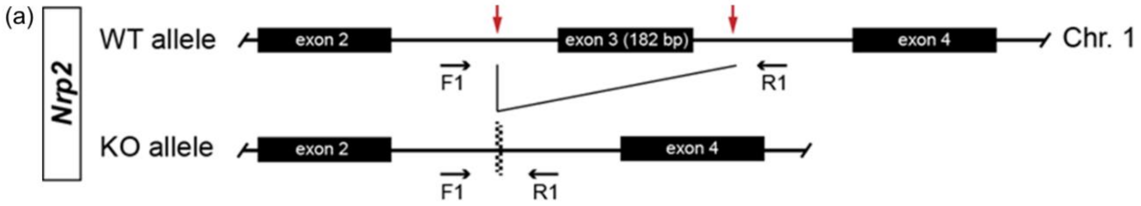

(b)

crRNA name	sequence (5´-3´)
Nrp2 sg1	TGTTACAGACGGATCAACAA
Nrp2 sg2	CCTGATATCATCCATCGACC

(c)

Primer name	sequence (5´-3´)
Nrp2_fwd	CACGTCATCATGAGGGAGCAG
Nrp2_rev	TCGCTCGACCTGCCTTTTATTA

CRISPR/Cas9-mediated generation of Nrp2-deficient MC3T3-E1 cells. (A) Schematic representation of the Nrp2 gene locus and targeting strategy for CRISPR/Cas9-mediated generation of Nrp2-deficient MC3T3-E1 cells. Targeting sites of guide RNAs are depicted by red arrows. Genotyping primer binding sites are depicted by black arrows. Nrp2-deficient MC3T3-E1 cells were generated by nucleofection of ribonucleoprotein (RNP) complexes using a 4D nucleofector system (Lonza, Cologne, Germany). For ribonucleoprotein (RNP) formation, crRNA (see (B) for sequence details) and tracrRNA (IDT, Leuven, Belgium) were combined in IDTE buffer (IDT) to a final concentration of 100 µM and annealed (95°C, 5 min; cool down to room temperature with -0,2°C/sec.). For RNP assembly, 21 µM high-fidelity Cas9 protein (IDT), and 12 µM of each cr/tracrRNA were combined in PBS and incubated for 15 min at room temperature. Nucleofection was performed using the Amaxa SE Cell Line 4D-Nucleofector X Kit (catalog-no. V4XC-1032, Lonza) according to the manufacturers instructions. Briefly, 1.5x105 cells were pelleted by centrifugation (800 rpm, 7 min) and resuspended in 20 µl Nucleofector SE solution. Cells were supplemented with 5 µl of RNP and 1 µl 100 µM Electroporation Enhancer (IDT). Nucleofection was performed using program CM-137. Subsequently, cells were resuspended in 70 µl pre-warmed culture medium, seeded (in one well of a 12 well-plate supplemented with 1 ml culture medium) and incubated for 48 h in a tissue culture incubator (37°C, 6% CO_2). To generate monoclonal Nrp2-deficient cell lines, single cells of the bulk population were seeded in 96-well plates by limiting dilution cloning. Established monoclonal cell lines were genotyped by PCR. PCR reactions were assembled according to the manufacturers protocol of the DreamTaq Green DNA Polymerase (EP0712, Thermo Fisher, Paisley, UK) using gene-specific primers listed in (C).

Original immunoblots (IB) related to Fig. S2 of osteodifferentiation in the presence (Nrp2 WT) or absence (Nrp2-KO) of neuropilin 2. Antibodies directed against Ncam1, Nrp2 or ß actin were used.

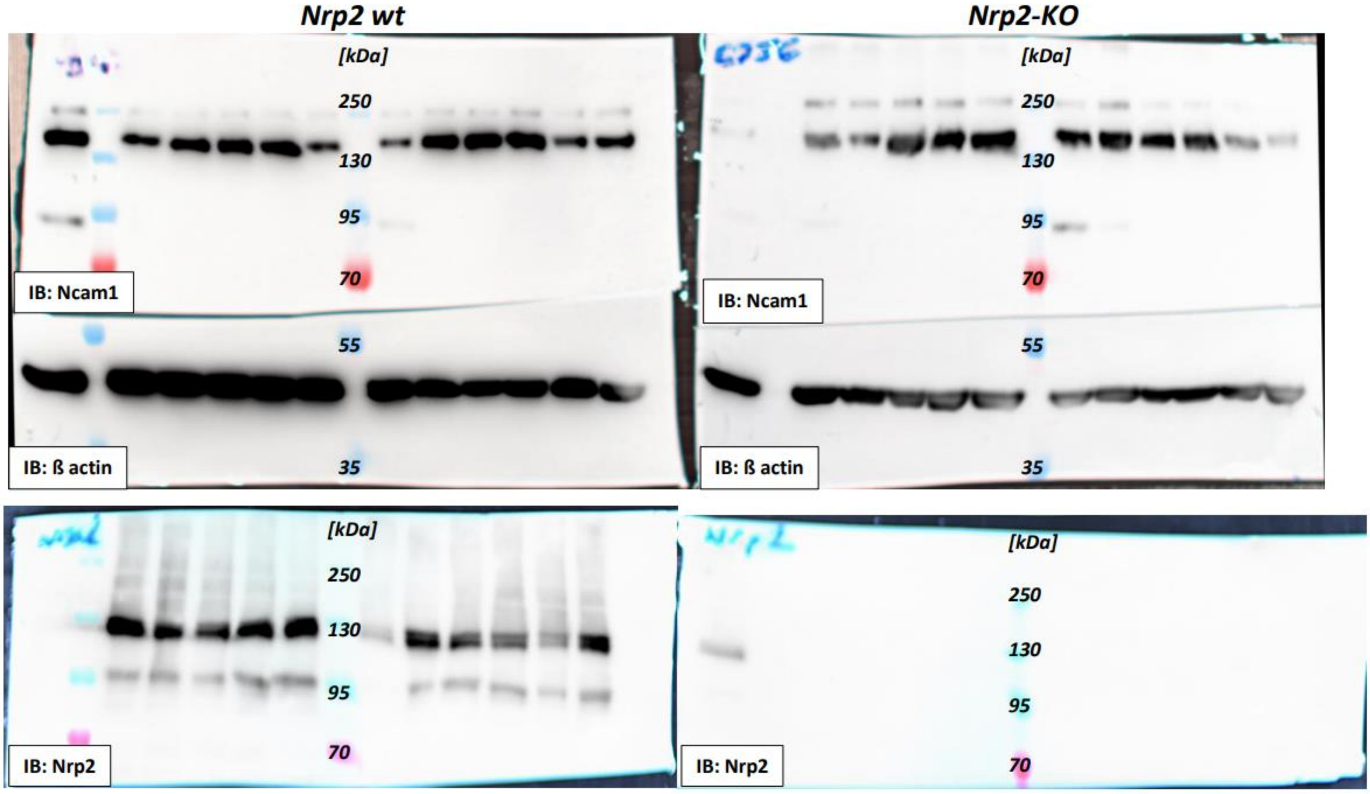

Supplement: Supplementary file 1 [file cancers-16-01924-s001.zip › Supplementary Materials TM (002)_KV_TM 21 05 2024.pdf]
